# Helicopter Emergency Medical Services Transport of COVID-19 Patients in the “First Wave”: A National Survey

**DOI:** 10.7759/cureus.16961

**Published:** 2021-08-06

**Authors:** Christopher L Berry, Monica C Corsetti, Francis Mencl

**Affiliations:** 1 Department of Emergency Medicine, Guthrie Robert Packer Hospital, Sayre, USA; 2 Department of Emergency Medicine, Penn State Health Milton S. Hershey Medical Center, Hershey, USA

**Keywords:** helicopter ems, covid-19, personal protective equipment (ppe), critical care transport, infection control, infection prevention and control

## Abstract

Study objectives

Helicopter emergency medical services (HEMS) providers have had to adjust to transporting patients with a novel, highly infectious pathogen. This study describes how HEMS organizations in the USA approached the coronavirus disease 2019 (COVID-19) pandemic in its first wave.

Methods

A survey was distributed via REDCap™ to HEMS organizations in May of 2020 using a national database. Data were collected regarding agency demographics and COVID-19 practices, including education, risk assessment, protective measures, equipment use, and decontamination practices. These were analyzed for qualitative observations and program attributes for COVID transport.

Results

A total of 68/287 (24%) organizations responded and completed the survey. Eighty-five percent (85%) of responding programs reported that they chose to transport known or suspected COVID-19 patients by air medical transport. Of responding programs, 93% provided education to their providers regarding COVID-19 and 100% conducted a COVID-19 risk analysis for patient transports. Of agencies transporting known or suspected COVID-19 patients, 77% required the use of N95 filtering facepiece respirators (N95) or powered air-purifying respirators (PAPR) for crewmembers during known or suspected COVID-19 patient transfers and 95% provided N95 respirators for pilots during transport. Five percent of responding programs utilized portable negative pressure isolation units. For COVID-19 transporting and non-transporting agencies, when transporting non-COVID-19 known or suspected patients, personal protective equipment (PPE) practice varied but tended to be more relaxed. Some services separated pilots from providers even during downtime (29%). Among services transporting known or suspected COVID-19 patients, the most common decontamination practice was manual wipe-down of all surfaces for a downtime of less than two hours.

Conclusion

The majority of survey respondents report that their programs chose to transport patients with known or suspected COVID-19 by air medical transport. However, there was high variability in practices regarding the transport of known or suspected COVID-19 as well as that of non-COVID-19 known or suspected patients by air during the initial outbreak of the pandemic. The HEMS industry may benefit from further research and standardization of airborne highly infectious disease transport practices in preparation for the next respiratory virus pandemic.

## Introduction

The coronavirus disease 2019 (COVID-19) pandemic has affected all health care providers. The air medical transport industry and helicopter emergency medical services (HEMS) programs have had to adjust to transporting patients with a novel, highly infectious pathogen. Many HEMS have adapted their practices to protect their crews, patients, and services. However, the severe acute respiratory syndrome coronavirus-2 (SARS-CoV-2) has created unprecedented challenges to helicopter transport given the confined space and limited resources available in transport, as well as the expense and expertise involved. The Centers for Disease Control (CDC) had previously provided guidelines for air medical transport during the 2003 severe acute respiratory syndrome (SARS) epidemic, including the use of N95 filtering facepiece respirators (N95) and high-efficiency particulate air (HEPA) filters for ventilator-dependent patients [1]. The Air Medical Physicians Association (AMPA) has recommended that HEMS providers be familiar with World Health Organization (WHO), local, regional, and national guidelines on transport and has recommended avoiding performing aerosol-generating procedures in confined spaces, which is often impossible in the HEMS setting [2]. Differences in local and regional practices invite variation in the way HEMS programs protect their crews. Some services have published their own guidelines and practices so that other services may learn from their experiences [3-5]. In addition, guidelines designed for fixed-wing transport have been described, introducing additional considerations and possible variations in practice [6].

The initial COVID-19 surge provided a new challenge for HEMS programs. With a lack of standardization in the industry across the United States with regard to equipment, airframes, availability of alternative transport modes, and program capabilities, we predicted that we would find a large variation in practice with regard to how HEMS programs chose to approach safe transport of known or suspected COVID-19. Previous publications have obtained qualitative data regarding COVID-19 infection prevention in smalls groups of HEMS programs [7], and there have been attempts to use data to model HEMS capabilities in the event of a COVID-19 surge [8]. Our study involved a survey of HEMS organizations to determine which protective measures are being adopted by transport services during the initial COVID-19 pandemic surge across a wide geography and variety of HEMS service types.

## Materials and methods

An institutional review board (IRB)-approved (Penn State Health Milton S. Hershey Medical Center IRB study ID: STUDY00015204, non-human subjects research) online survey was distributed to HEMS programs using a national database: The Association of Air Medical Services 2019 Publication Atlas was used to identify participants. This list included 303 HEMS organizations; 288 of which provided an email contact. The survey was distributed in May 2020 to program directors/managers or medical directors via individual invitations using the provided emails. For non-responders, the survey was sent a total of four times; the second on day 5, the third on day 12, and the fourth on day 19. When the email was returned as undeliverable, the authors made efforts to reach out to the organization by other electronic mail addresses. Each organization had a unique survey link distributed to them to prevent any organization from submitting greater than one response. Submission of the online survey constituted written consent for inclusion in the study.

Basic HEMS program information was collected, including location by state, the number of annual transports, and service model classification. Descriptive statistics were collected regarding COVID-19 practices, including patient risk assessment, personal protective equipment (PPE), patient packaging/treatment, and decontamination practices (please see Appendix). Results reported are rounded off and may not always add up to 100%.

Study data were collected and managed using REDCap™ electronic data capture tools hosted at Penn State Health [9-10]. REDCap (Research Electronic Data Capture, Vanderbilt University) is a secure, web-based software platform designed to support data capture for research studies providing 1) an intuitive interface for validated data capture; 2) audit trails for tracking data manipulation and export procedures; 3) automated export procedures for data integration and interoperability with external sources. This information was accessed and analyzed by project authors. Statistical analysis software Minitab™ (Minitab, LLC, State College, Pennsylvania) [11] and SAS System Statistical Analysis (SAS Institute, Cary, North Carolina) [12] were used to determine statistical significance between COVID-19-transporting services and non-COVID-19 transporting services.

Descriptive statistics were generated including means, medians, and standard deviations for continuous variables. Comparisons between categorical variables were analyzed using contingency table analysis; significance levels were determined by chi-squared tests and Fisher's exact tests. No adjustments for multiple tests were made. All tests were two‐sided and based on a significance criterion of p <0.05.

For convenience, we included suspected cases with known COVID-19-positive patients. Information was collected about various service attributes in order to determine the correlation between the decisions to transport COVID-19 patients. These included the number of bases, yearly number of transports, service classification, instrument flight rules (IFR) capabilities, night vision goggle (NVG) capabilities, extracorporeal membrane oxygenation (ECMO) capabilities, and ability to transport critical care patients by ground units. A chi-squared test for association and Fisher’s exact test were used to analyze responses between known and suspected COVID-19 transporting programs and those not transporting suspected COVID-19 cases for statistical significance in a number of bases, yearly transports, and service types. A two-sample proportion test and Fisher’s exact test were used to determine statistical significance between COVID-19 transporting facilities and those not transporting suspected COVID-19 cases in terms of IFR capabilities, night vision capabilities, ECMO capabilities, and ability to transport critical patients by ground units. All respondents were surveyed about the education of crews regarding COVID-19 and screening processes.

Responses were further categorized by the ability to transport COVID-19-suspected patients versus those services unable to do so. Qualitative responses were analyzed to gain information about common decontamination procedures, personal protective equipment practices for pilots and crew members, equipment and safety techniques utilized in transport, and other metrics with an aim for the general description of the current operating state of the service. For those services that were unable to transport COVID-19 patients, descriptive data are further gathered, including the type of PPE and equipment utilized, screening processes, and decontamination procedures.

## Results

**Figure 1:**
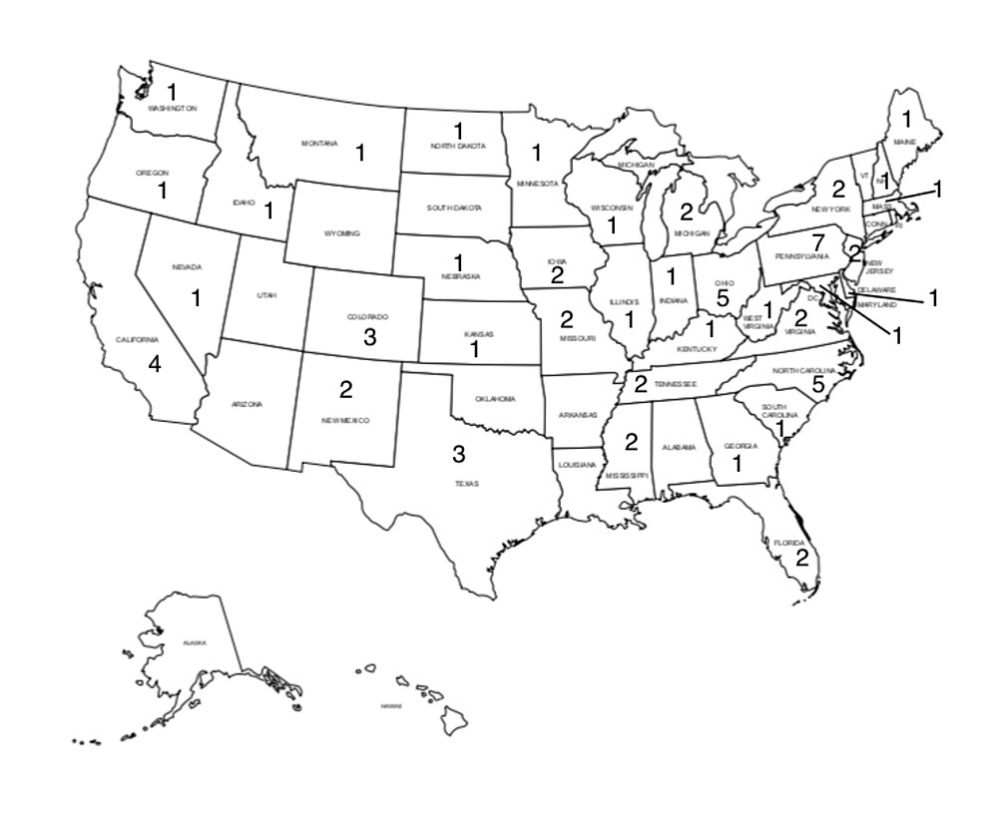
State Map of Responding Programs (By Primary State Served) https://www.freeusandworldmaps.com/html/USAandCanada/USPrintableNoText.html

A total of 68 HEMS organizations responded, representing a 24% response rate. The primary state that each service was recorded in and this distribution is depicted in Figure 1. In total, 58 organizations out of 68 (85%) that completed the survey responded that they transported known and/or suspected COVID-19 cases by helicopter and/or fixed-wing at the time of the survey. Of these, 52% stated that these composed 0-10% of their total flights in April 2020 while 45% stated that they had 0-10 flights with COVID-19 cases in total (Table 1).

**Table 1:** Service Demographics

Number of Transport Bases	(% of Total)	COVID Transporting (% Transporting)	Non-COVID Transporting (% Non-Transporting)
1 Base	24 (35%)	18 (31%)	6 (60%)
2-3 Bases	21 (31%)	18 (31%)	3 (30%)
4-5 Bases	12 (18%)	11 (19%)	1 (10%)
6-10 Bases	8 (12%)	8 (14%)	0
>10 Bases	3 (4.4%)	3 (5.2%)	0
Total Yearly Transport Flights			
<500	21(32%)	15 (26%)	6 (60%)
501-750	6 (9%)	5 (8.6%)	1 (10%)
751-1000	6 (9%)	6 (10%)	0
1001-1250	7 (11%)	5 (8.6%)	2 (20%)
1251-1500	7 (11%)	6 (10%)	1 (10%)
>1500	19 (29%)	19 (33%)	0
Service Type			
Private	11 (16%)	11 (19%)	0
Police-Based	3 (4.4%)	0	3 (30%)
Hospital-Based	46 (68%)	40 (69%)	6 (60%)
Other	8 (12%)	7 (12%)	1 (10%)
Crew Configurations			
Nurse/Medic	50 (73.5%)	44 (76%)	6 (60%)
Nurse/Resp Therapist	3 (4.4%)	3 (5.2%)	0
Nurse/Physician	1 (1.5%)	1 (1.7%)	0
Nurse/Nurse	8 (12%)	8 (14%)	0
APP/Medic	1 (1.5%)	1 (1.7%)	0
Medic/Medic	4 (6%)	1 (1.7%)	3 (30%)
Medic/EMT-B	1 (1.5%)	0	1 (10%)
Fixed-Wing Aircraft Utilization			
Yes	29 (43%)	26 (45%)	3 (30%)
No	39 (57%)	32 (55%)	7 (70%)
Dedicated Critical Care Ground Unit Availability			
Yes	51 (75%)	44 (76%)	7 (70%)
No	17 (25%)	14 (24%)	3 (30%)
Instrument Flight Rules (IFR) Capability			
Yes	47 (69%)	43 (74%)	4 (40%)
No	21 (31%)	15 (26%)	6 (60%)
Night Vision Goggles (NVGs)/Low Light Operations Capabilities?			
Yes	64 (94%)	55 (95%)	9 (90%)
No	4 (5.9%)	3 (5.2%)	1 (10%)
Extracorporeal Membrane Oxygenation (ECMO) Capabilities			
Yes	41 (60%)	37 (64%)	4 (40%)
No	27 (40%)	21 (36%)	6 (60%)

The largest proportion of responding programs (35%) had only one base, and only 4% of services had greater than 10 bases (Table 1). Thirty-two percent of flight services flew fewer than 500 transports per year while 29% did more than 1500 flights annually. Most services described themselves as hospital-based (68%), fewer as private services (16%), while 12% described themselves as something other than the choices listed, including a “State Police state-funded hospital partnership” and “Hospital consortium”. Seventy-three percent of services utilized a nurse/medic crew configuration, 12% flew with two nurses, and only two (3%) had physicians on board. Three-quarters of the services also have dedicated ground transport, but only slightly more than one-third (36%) also used fixed-wing in their operations. The majority have IFR capability (69%) and almost all have NVG capability (94%).

There was no statistically significant difference in whether a service responded whether they did or did not transport cases of COVID-19 based on the number of bases (p=0.305) or the number of yearly transports (p=0.135). There was a suggestion of a correlation between transport service type and whether a service provided known or suspected COVID-19 transport (p<0.001). Notably, there is a suggestion that police-based systems have a negative correlation for transporting known or suspected COVID-19 patients while private services have a positive correlation; however, the study is not sufficiently powered to detect these differences. A two-sample proportion test and Fisher’s exact test showed no statistically significant correlation between COVID-19 transporting and non-COVID-19 transporting services for fixed-wing transport (p=0.498), ground transport capabilities for critical care patients (p=0.702), IFR capabilities (p=0.058), NVG/low light capabilities (p=0.479), ECMO capabilities (p=0.179), or IFR capabilities (p= 0.058).

The majority of programs, including 90% of non-transporting and 93% of transporting services, provided some type of education for their crews regarding COVID-19 symptoms or lab/radiologic abnormalities (Table 2). All services screen for COVID-19, with the most common screening in both transport and non-transport groups being conducted by air medical dispatchers prior to dispatch. Both groups most heavily relied on patient clinical history and presentation, chest imaging, and the transferring physician’s report during COVID-19 screening (Table 2).

**Table 2:** COVID-19 Medical Crew Education and Risk Analysis

	All Responses (% of Total)	Transporting (% of Transporting)	Non-Transporting (% of Non-Transporting)
Services Provided Training to HEMS Crews About the Clinical Presentation of COVID-19 Patients and Common Radiographic/Laboratory Values Suspicious of COVID-19			
Yes	63 (93%)	54 (93%)	9 (90%)
No	4 (5.9%)	4 (6.9%)	0
Time at which services screen for COVID-19 during patient transfers			
By air medical dispatchers prior to dispatch	58 (85%)	52 (90%)	6 (60%)
By HEMS crew prior to dispatch	14 (21%)	13 (22%)	1 (10%)
By HEMS crew after dispatch	12 (18%)	9 (16%)	3 (30%)
By Medical Director prior to dispatch	10 (15%)	10 (17%)	0
By accepting physician prior to dispatch	17 (25%)	13 (22%)	4 (40%)
By HEMS crew on arrival to transferring facility	41 (60%)	36 (62%)	5 (50%)
Not screening for COVID-19 patients	0	0	0
Other	7 (10%)	6 (10.3%)	1 (10%)
Data Used by Crews to Make Risk Assessment Patients’ COVID-19 Status			
Transferring physician's report	55 (81%)	50 (86%)	5 (50%)
Radiographs including chest X-ray, CT scan	32 (47%)	31 (53%)	1 (10%)
COVID-19 testing results	55 (81%)	50 (86%)	5 (50%)
Laboratory values	18 (26%)	18 (31%)	0
Patient Clinical History/Presentation	58 (85%)	50 (86%)	8 (80%)
Not making a COVID-19 risk assessment	3 (4.4%)	2 (3.4%)	1 (10%)
Other	5 (7.4%)	5 (8.6%)	0

The most frequently used PPE strategy by COVID-19-transporting programs was the use of an N95 mask at all times, with 76% of these services requiring this of their patient care personnel (Table 3). Among programs transporting known or suspected COVID-19 patients, for transports not involving a COVID-19 case, the most commonly required respiratory PPE was surgical mask only (60%), followed by an N95 at all times (26%). With regards to contact PPE, for COVID-19 transports, 98% of services required gloves (vs. 95% for non-COVID-19 transports), 100% required face shields or goggles (vs. 55% for non-COVID-19 transports), and 81% required a surgical gown (vs. 14% for non-COVID transports), while only 29% required disposable coveralls (5% for non-COVID-19 transports) (Table 3).

**Table 3:** Number of Services Requiring Each PPE Type Among COVID-Transporting and Non-Transporting Services

PPE Required for Patient Care Personnel	For Suspected/Positive COVID-19 Flights (% of Transporting Services)	For Flights With No Suspicion for COVID-19 but on a Transporting Service (% of Transporting Services)	For Non-Transporting Services (% of Non-Transporting Services)
Respiratory PPE			
Surgical mask	2 (3.4%)	35 (60%)	5 (50%)
N95 mask at all times	44 (76%)	15 (26%)	3 (30%)
N95 mask for aerosolized procedures only	6 (10%)	4 (6.9%)	6 (60%)
Purified air-powered respirator (PAPR) at all times	1 (1.7%)	0	0
PAPR for aerosolized procedures only, surgical mask otherwise	0	0	1 (10%)
Other	5 (8.6%)	4 (6.9%)	1 (10%)
Contact PPE			
Gloves	57 (98%)	55 (95%)	9 (90%)
Face shields/goggles	58 (100%)	32 (55%)	6 (60%)
Surgical gown	47 (81%)	8 (14%)	7 (70%)
Disposable protective coveralls (bunny suit)	17 (29%)	3 (5.2%)	0
Other	5 (8.6%)	4 (6.9%)	2 (2%)

Programs transporting known or suspected COVID-19 cases responded that 95% provided their pilots with N95 masks while none used a PAPR (Table 4). Twenty-six percent of all services (including non-transporting) separated the pilot from the crew while not transporting a patient. A majority of services prohibit the pilot from entering referring facilities and most services (including half of those not transporting COVID-19 cases) limit pilots from participating in activities such as loading the patient or interacting with the patient and family (Table 5).

**Table 4:** Level of Respiratory PPE Provided to Pilots by Services

	Number of Transporting Services Utilizing Intervention in COVID-19 cases (% of Transporting Services)	Number of Non-Transporting Services Utilizing Intervention in (% of Non-Transporting Services)
None	1 (1.7%)	0
Surgical Mask	24 (41%)	5 (50%)
N95 Mask	55 (95%)	9 (90%)
Other	3 (5.1%)	1 (10%)

**Table 5:** COVID-19 Pilot Isolation Protocols

	All Responses (% of Total)	Transporting (% of Transporting)	Non-Transporting (% of Non-Transporting)
Separation of Pilots from Patient Care Crew When Not on Mission			
Yes	18 (26%)	15 (26%)	3 (30%)
No	50 (74%)	43 (74%)	7 (70%)
Activities in Which Pilots’ Participation is Limited			
Loading Patient Into Airframe	50 (74%)	45 (78%)	5 (50%)
Interacting With Patient or Patient Family	48 (71%)	43 (74%)	5 (50%)
Entering Referring Facility	46 (68%)	40 (69%)	6 (60%)
None of the Above	12 (18%)	9 (16%)	3 (30%)

Finally, 29 services transporting COVID-19 cases (50%) stated that their rotor-wing transport allowed the pilot to be sealed/isolated from the patient compartment, and out of the 26 services that both transport known and suspected COVID-19 patients and have fixed-wing transport, 12 (46%) responded that their airframes allow the pilot to be sealed/isolated from the patient compartment.

Approximately 91% of programs indicated that they transport non-intubated known or suspected COVID-19 patients. However, fewer report transporting these patients with potentially aerosolizing measures during transport, with 29% completing transports with a high-flow nasal cannula (HFNC) and 28% with non-invasive positive pressure ventilation (NIPPV). A majority of services transport patients on a nasal cannula and non-rebreather (78% and 76%, respectively). Other interventions, such as ECMO or proning, were less common (Table 6).

**Table 6:** Patient Packaging Practices and Considerations for Services Transporting COVID-19/PUIs BIPAP: Bilevel Positive Airway Pressure; CPAP: Continuous Positive Airway Pressure; PUI: Patient Under Investigation

	Number of Services Planning to Utilize Intervention
Transporting Non-Intubated COVID-19 Positive/Suspected Patients	53 (91%)
Interventions Utilized in Transport	
High-Flow Nasal Cannula	17 (29%)
Non-Invasive Ventilation (Incl. BIPAP or CPAP)	16 (28%)
Nasal Cannula	45 (78%)
Non-Rebreather	44 (76%)
Using Portable Negative Pressure Isolation Units (IE an "adult isolette")	3 (5.2%)
Proned Positioning	15 (26%)
ECMO	6 (10%)

By far the most commonly used method of decontamination was manual surface wipes (95%), followed by germicidal spray (67%) and chlorine dioxide (2%) (Table 7). Most services reported downtime of <2 hours (Table 8). Rarer methods of cleaning such as ultraviolet (UV) irradiation, chlorine dioxide, and hydrogen peroxide were analyzed; UV irradiation reported a shorter-than-average downtime, and chlorine dioxide and hydrogen peroxide yielded longer-than-average downtimes.

**Table 7:** Decontamination Methods for Airframes Post-COVID-19/PUI Transport UV: Ultraviolet; PUI: Patient Under Investigation

Method	Number of Programs Using (% of Transporting)	Number of Programs Using (% of Non-Transporting)
Manual Surface Wipes	55 (95%)	9 (90%)
Sprayed Germicidal Disinfectant	39 (67%)	5 (50%)
UV Light Germicidal Irradiation	7 (12%)	0
Chlorine Dioxide Gas (IE Aeroclave System)	1 (1.7%)	1 (10%)
Hydrogen Peroxide Vapor	4 (6.9%)	0
Other	7 (12%)	2 (20%)

**Table 8:** Average Downtime Post-Transport

Downtime	Number of Transporting Services Post COVID-19 Transport (% of Transporting)	Number of Non-Transporting Services (% of Non-Transporting)
<1 hr	28 (48%)	7 (70%)
1 to 2 hrs	25 (43%)	2 (20%)
2 to 3 hrs	3 (5.1%)	0
> 3 hrs	1 (1.7%)	1 (10%)
N/A	1 (1.7%)	7 (70%)

Ten respondents indicated that their services would not transport any patients with suspected or confirmed COVID-19 patients. Of these services, 70% felt they did not have the ability to adequately protect their pilots from infection,50% listed increased downtime or decontamination as an issue, and 20% noted that they did not have an adequate supply of PPE to safely provide care (Table 9). Other comments noted included a limited pool of pilots with a limited ability to stay in service if any were to fall ill.

**Table 9:** Factors Leading to Program Decision Not To Transport COVID-19 Patients by HEMS HEMS: Helicopter Emergency Medical Services

	Number of Services Listing This as a Factor
Inability to protect flight crew (IE Pilot)	7 (70%)
Inability to protect patient care crew	3 (30%)
Lack of PPE supply	2 (20%)
Increased airframe downtime/unavailability due to decontamination process	5 (50%)
Other	4 (40%)

Within the 10 services indicating that they do not plan to transport known or suspected COVID-19 patients, fewer (50%) services required their crew to wear a surgical mask when compared to the transport of non-COVID-19 patients by services that do routinely transport COVID-19 patients (60%). The N95 mask mandates at all times were higher for these services (30%) than during non-COVID-19 transports of COVID-19-transporting programs (26%). N95s and PAPR use for aerosolizing procedures were notably higher in this group than during non-COVID-19 transports of COVID-19-transporting capable programs, with N95 use at 60% vs 7%, respectively, and PAPR use at 10% vs 0%, respectively (Table 3).

With regards to contact isolation in non-COVID-19-transporting programs, 90% of patient care personnel were required to wear gloves at all times vs. 95% of patient care personnel on non-COVID-19 transports of COVID-19-transporting capable programs. Among non-COVID-19 transporting programs, 70% required their personnel to wear gowns for transports during the pandemic period vs. 14% of non-COVID-19 transports of COVID-19-transporting capable services (Table 3).

Ninety percent of pilots for non-transporting programs were given N95 masks vs. 95% in COVID-19 transporting services (Table 4). One service commented that they were moving to a P100 particulate respirator model for pilots.

Finally, 90% of respondents from non-COVID-19-transporting programs utilized manual surface wipes for decontamination while 50% utilized germicidal disinfectant for decontamination (Table 7). The downtime was on average less than two hours in the non-COVID-19-transporting group (Table 8).

## Discussion

The COVID-19 pandemic has posed a significant challenge for HEMS programs. Protecting medical and flight crews from a novel, highly infectious respiratory pathogen, such as SARS-CoV-2, particularly early in the pandemic without significant data regarding its transmission, required HEMS programs to make multiple operational decisions on how they would protect their crews and continue to deliver care. Among these decisions include how they would identify patients who were suspected of COVID, how they would protect their crew members, and whether they would agree to transport COVID-19 patients at all. In May 2020, this was being done in an environment with a paucity of reliable data regarding the symptoms, transmission, and best treatment practices for SARS-CoV-2 in the HEMS environment. The 2003 SARS pandemic had been relatively geographically contained and short-lived, although some literature was produced for future guidance [1].

This survey was contrived to obtain both quantifiable and qualitative data to characterize how HEMS programs responded to COVID-19 early in the pandemic. Approximately 85% of responding programs planned to transport confirmed or suspected COVID-19 patients. It could be argued, however, that despite not knowingly taking COVID-19 suspected or positive patients, any patient during the pandemic could potentially have been an infectious risk. COVID-19 has been reported to have a variable asymptomatic rate between 1.6% and 56.5 % [13] Therefore, even programs that decline to complete transfers of COVID-19 patients by rotor-wing or fixed-wing transports have a strong likelihood of transporting an asymptomatic, yet infected, patient. It is not surprising that the majority of programs reported taking steps to protect their providers and flight crews, such as requiring respiratory protection at all times while providing patient care, whether knowingly transporting COVID-19 positive or suspected patients or not.

It should also be noted that at this point in the pandemic, unprotected exposure to a COVID-19 patient might lead to a quarantine period of several weeks and that a program may be at risk of not having enough personnel to continue operations due to quarantines alone, much less contract the disease.

The HEMS environment poses a number of potential challenges with regard to reducing transmission to crewmembers. Simulation has shown that aerosols readily distribute around an airframe, landing on all surfaces, including the avionics in non-separated cockpits [14]. While a negative pressure environment may be possible in a hospital or ground ambulance, many of the popular airframes used by HEMS programs are not designed to take the extra weight of a portable negative pressure isolation unit (or an "adult isolette"). Not surprisingly, even among responding programs transporting COVID-19 patients, our survey did not find the use of these to be common. This makes transporting patients undergoing potentially aerosolizing procedures, such as breathing treatments, HFNC, or NIPPV, a significant challenge in this environment. Furthermore, depending on airframe configuration, the patient may be positioned next to the pilot, precluding isolation of the pilot from the patient compartment, leading to the requirement for respiratory protection for the pilot. A respirator on a pilot may pose some challenging questions. Does the respirator cause the pilot's goggles to fog? Does it reduce the pilot's or medical crews' ability to communicate by radio? Some of the non-COVID-19-transporting programs responding note the difficulty with protecting pilots as a cause in their decision to not transport known or suspected COVID-19 patients early in the pandemic. It remains to be seen if the HEMS industry will move towards adapting more capable airframes in lieu of these challenges.

Airframe decontamination poses a challenge in itself. If aerosols are spreading throughout the inside of the airframe [14], how best to remove them? Decontamination methods, such as surface wipes and sprayed germicidal agents, were more commonly reported. More thorough decontamination methods, such as UV decontamination, were less commonly reported. The use of chemical disinfectants on avionics surfaces is limited due to the risk of decontamination, and it is possible that the industry will need to adapt to use other methods, such as UV decontamination, to successfully remove pathogens [15].

Limitations

This study has several limitations, most notably a low response rate and sampling/non-response bias. An email survey poses challenges in regard to the motivation of respondents. In April 2020, the pandemic in the United States was relatively localized in urban centers, and some parts of the country may not have had to deal with the realities of a high burden of COVID-19 patients in their local system. The survey does provide qualitative information for a variety of topics relevant to how HEMS organizations have responded to the COVID-19 pandemic and provides insight into some of the challenges facing these organizations. Furthermore, our data represent a cross-section of data while the pandemic continues to evolve. Future research should focus on determining how these services changed their practices in the face of a sustained challenge and the effects of the pandemic on staffing, finances, and resources.

## Conclusions

Early in the COVID-19 outbreak, the majority of HEMS programs chose to transport known or suspected COVID-19 patients by air. The majority of programs took multiple steps to modify their practice, including educating crew regarding COVID-19, implementing screening practices for known or suspected COVID-19 patients, modifying patient packaging and treatment interventions, and adjusting decontamination practices and PPE use to protect their crews. However, there was variation in practice. Further study is needed to examine the impact of a sustained pandemic on these practices, and particularly whether they may have relaxed as effective vaccinations have become more prevalent. Given the observed variation, the development of universal guidance for the safe transport of highly infectious airborne pathogens in the HEMS environment would be of benefit.

## Appendices

Survey questions

Section A: Program Demographics

1. What state/territory is your program primarily based out of?

o   Alabama

o   Alaska

o   Arizona

o   Arkansas

o   California

o   Colorado

o   Connecticut

o   Delaware

o   Florida

o   Georgia

o   Hawaii

o   Idaho

o   Illinois

o   Indiana

o   Iowa

o   Kansas

o   Kentucky

o   Louisiana

o   Maine

o   Maryland

o   Massachusetts

o   Michigan

o   Minnesota

o   Mississippi

o   Missouri

o   Montana

o   Nebraska

o   Nevada

o   New Hampshire

o   New Jersey

o   New Mexico

o   New York

o   North Carolina

o   North Dakota

o   Ohio

o   Oklahoma

o   Oregon

o   Pennsylvania

o   Rhode Island

o   South Carolina

o   South Dakota

o   Tennessee

o   Texas

o   Utah

o   Vermont

o   Virginia

o   Washington

o   West Virginia

o   Wisconsin

o   Wyoming

o   Washington DC

o   Puerto Rico

o   U.S. Virgin Islands

o   American Samoa

o   Guam

o   Northern Mariana Islands

o   Baker Island

o   Howland Island

o   Jarvis Island

o   Johnston Atoll

o   Midway Islands

o   Wake Island

o   Palmyra Atoll

o   Kingman Reef

o   Navassa Island

o   Serranilla Bank

o   Bajo Nuevo Bank

2. How many Helicopter Emergency Medical Services (HEMS)/air transport bases do you have?

o   1

o   2-3

o   4-5

o   6-10

o   Greater than 10

3. Which of the following best describes your service?

o   Private

o   Military

o   Police Based

o   Hospital Based

o   Charity

o   Municipal

o   Other (Specify)

o   If you selected other above, please specify

4. What was your system's total volume of HEMS/air transport flights last year?

o   Less than 500

o   501 - 750

o   751 - 1000

o   1001 - 1250

o   1251 - 1500

o   Greater than 1500

5. What rotor wing airframes does your service utilize? (Select all that apply)

o   Bell 206/407

o   Bell 222/230

o   Bell 212

o   Bell 412

o   Bell 427

o   Bell 429

o   AS 332/EC 225 Super Puma

o   AS 350/H125

o   AS 355

o   AS 365 Dauphin

o   AS 565 Panther

o   EC 130/H 130

o   EC 135/H 135

o   EC 145/H 145

o   EC 155/H 155

o   AW 109

o   AW 139

o   Bo 105

o   BK 117

o   MD 902

o   Sikorsky S 76

o   Sikorsky S/UH 70

o   Sikorsky S 92

o   Mil Mi-8

o   Kazan Ansat - M

o   Other (Specify)

o   If you answered other above, please specify

6. Does your service utilize fixed wing aircraft?

o   Yes

o   No

7. Which fixed wing aircraft does your service utilize? (Fill in the blank)

8. Does your program have the ability to transport patients requiring critical care treatment by a dedicated critical care ground unit?

o   Yes

o   No

9. Does your program have the capability to fly via Instrument Flight Rules (IFR)?

o   Yes

o   No

10. Does your program have the capability to fly with night vision goggles (NVGs)/low light operations?

o   Yes

o   No

11. Does your service have the ability to transport patients on Extracorporeal Membrane Oxygenation (ECMO)?

o   Yes

o   No

12. What is your most common crew configuration?

o   Nurse/Medic

o   Nurse/Respiratory Therapist

o   Nurse/Physician

o   Nurse/Nurse

o   Physician/Medic

o   Advanced Practice Provider (IE, Physician Assistant/Nurse Practitioner)/Medic

o   Medic/Medic

o   Other (Specify)

Section B - Program COVID-19 Risk Analysis

1. Have you provided specific training to your HEMS crews about the clinical presentation of COVID-19 patients and common radiographic/laboratory values which would be a cause for suspicion of COVID-19?

o   Yes

o   No

2. At what time are you screening for COVID-19 patients during patient transfers? (select all that apply):

o   By air medical dispatchers prior to dispatch

o   By HEMS crew prior to dispatch

o   By HEMS crew after dispatch

o   By Medical Director prior to dispatch

o   By Accepting physician prior to dispatch

o   By HEMS crew on arrival to transferring facility

o   We are not screening for COVID-19 patients

o   Other (Specify)

3. What data are your crews using to make their risk assessment for the patient's COVID-19 status? (select all that apply):

o   Transferring Physician's Report

o   Radiographs Including Chest X-ray, CT scan

o   COVID-19 testing results

o   Laboratory Values

o   Patient Clinical History/Presentation

o   We are not making a COVID-19 risk assessment

o   Other (Specify)

4. Are you transporting patients who are under suspicion of having or confirmed as having COVID-19?

o   Yes - by rotor wing ONLY

o   Yes - by fixed wing ONLY

o   Yes - by rotor wing AND fixed wing

Section C, D, E: Programs Transporting COVID-19 Patients by Air Page 1/4

1. What percentage of flights completed by your program in April, 2020 were for COVID-19 patients?

o   0-10

o   11-20

o   21-30

o   31-40

o   41-50

o   51-60

o   61-70

o   71-80

o   81-90

o   91-100

o   Information not available at this time

2. What was the number of flights in April completed by your program for COVID-19 patients?

o   0-10

o   11-20

o   21-30

o   31-40

o   41-50

o   51-60

o   61-70

o   71-80

o   81-90

o   Greater than 100

o   Information not available at this time

Section C, D, E: Programs Transporting COVID-19 Patients by Air Page 2/4

3. What level of respiratory PPE are you requiring your patient care personnel wear for patient flights WITH suspicion for or confirmed COVID-19? (Select the options which best fit your program)

o   Surgical Mask

o   N95 mask at all times

o   N95 mask for aerosolized procedures only, surgical mask otherwise

o   Purified Air Powered Respirator (PAPR) at all times

o   Purified Air Powered Respirator (PAPR) for aerosolized procedures only, surgical mask otherwise

o   Other (Specify)

4. What level of respiratory PPE are you requiring your patient care personnel wear for patient flights WITHOUT suspicion for or confirmed COVID-19? (Select the options which best fit your program)

o   None

o   Surgical Mask

o   N95 mask at all times

o   N95 mask for aerosolized procedures only, surgical mask otherwise

o   Purified Air Powered Respirator (PAPR) at all times

o   Purified Air Powered Respirator (PAPR) for aerosolized procedures only, surgical mask otherwise

o   Other (Specify)

5. What level of contact PPE are you requiring your patient care personnel wear for patient flights WITH suspicion for or confirmed COVID-19 (Select all that Surgical Gown apply)?

o   Gloves

o   Face shield/goggles

o   Disposable Protective Coveralls ("Bunny Suit")

o   Other (Specify)

6. What level of contact PPE are you requiring your patient care personnel wear for patient flights WITHOUT suspicion for or confirmed COVID-19 (Select all that apply)?

o   Gloves

o   Face shield/goggles

o   Surgical Gown

o   Disposable Protective Coveralls ("Bunny Suit")

o   Other (Specify)

7. What level of respiratory PPE are you providing your pilots during the COVID-19 pandemic?

o   None

o   Surgical Mask

o   N95 mask

o   Purified Air Powered Respirator

o   Other (Specify)

Section C, D, E: Programs Transporting COVID-19 Patients by Air Page 3/4

8. Are you transporting non-intubated COVID-19 positive/suspected patients?

o   Yes

o   No

9. If you answered "Yes" above, are you transporting by HEMS patients with any of the following (Select all that apply):

o   High Flow Nasal Cannula

o   Non-Invasive Ventilation (including Bilevel Positive Airway Pressure [BIPAP] or Continuous Positive Airway Pressure [CPAP]

o   Nasal Cannula

o   Non-Rebreather Mask

10. Is your program packaging COVID-19 patients using Portable Negative Pressure Isolation Units (IE an "adult isolette")?

o   Yes

o   No

11. Has your program transported patients COVID-19 patients in a prone position?

o   Yes

o   No

12. Has your program transported COVID-19 patients who are currently on ECMO by air?

o   Yes

o   No

13. Explain any changes to patient packaging that you have made for COVID-19 patients: (Fill in the blank)

Section C, D, E: Programs Transporting COVID-19 Patients by Air Page 4/4

14. By which method are you decontaminating your airframes post COVID-19 patient transport (select all that apply)?

o   Manual Surface Wipes

o   Sprayed Germicidal Disinfectant

o   Ultraviolet Light Germicidal Irradiation

o   Chlorine Dioxide Gas (IE AeroClave System)

o   Hydrogen Peroxide Vapor

o   Other (Specify)

15. What is your average airframe down time after a COVID-19 patient transfer?

o   Less than 1 hour

o   Between 1 and 2 hours

o   Between 2 and 3 hours

o   Greater than 3 hours

16. Do the rotor wing airframes you are transporting COVID-19 patients with allow the pilot to be sealed/isolated from the patient compartment?

o   Yes

o   No

17. Do the fixed wing airframes you are transporting COVID-19 patients allow the pilot to be sealed/isolated from the patient compartment?

o   Yes

o   No

Section F: Pilot Considerations

1. Are you separating your pilots from your patient care crew when not completing a mission?

o   Yes

o   No

2. Are you limiting your pilots from participating in any of the following activities (Select all that apply):

o   Loading patient into the airframe

o   Interacting with patient or patient family

o   Entering the referring facility

o   None of the above

Section G: Non-Transporting Programs

1. What factors led to your program to decide not to transport COVID-19 patients by HEMS (Select all that apply)?

o   Inability to protect flight crew (IE pilot)

o   Inability to protect patient care crew

o   Lack of Personal Protective Equipment Supply

o   Increased airframe downtime/unavailability due to decontamination process

o   Other (Specify)

2. What level of respiratory PPE are you requiring your patient care personnel wear for patient flights during the ongoing COVID-19 pandemic? (Select the options which best fit your program)

o   None

o   Surgical Mask

o   N95 mask at all times

o   N95 mask for aerosolized procedures, surgical mask otherwise

o   Purified Air Powered Respirator (PAPR) at all times

o   Purified Air Powered Respirator (PAPR) for aerosolized procedures, surgical mask otherwise

o   Other (Specify)

3. What level of contact PPE are you requiring your patient care personnel wear for patient flights during the ongoing COVID-19 pandemic (Select all that apply)?

o   None

o   Gloves

o   Face shield/goggles

o   Surgical Gown

o   Disposable Protective Coveralls ("Bunny Suit")

o   Other (Specify)

4. What level of respiratory PPE are you providing your pilots during the COVID-19 pandemic?

o   None

o   Surgical Mask

o   N95 mask

o   Purified Air Powered Respirator

o   Other (Specify)

5. By which method are you decontaminating your airframes post patient transport during the ongoing COVID-19 pandemic (select all that apply)?

o   Manual Surface Wipes

o   Sprayed Germicidal Disinfectant

o   Ultraviolet Light Germicidal Irradiation

o   Chlorine Dioxide Gas (IE AeroClave System)

o   Hydrogen Peroxide Vapor

o   Other (Specify)

6. What is your average airframe down time after a patient transfer?

o   Less than 1 hour

o   Between 1 and 2 hours

o   Between 2 and 3 hours

o   Greater than 3

Section H: Comments

1. Are there any comments that you would like to note about your program's approach to the COVID-19 pandemic? (Please Specify)
